# A chromosomal rearrangement in a child with severe speech and language disorder separates *FOXP2* from a functional enhancer

**DOI:** 10.1186/s13039-015-0173-0

**Published:** 2015-08-20

**Authors:** Martin Becker, Paolo Devanna, Simon E. Fisher, Sonja C. Vernes

**Affiliations:** Max Planck Institute for Psycholinguistics, PO Box 310, Nijmegen, 6500 AH The Netherlands; Donders Institute for Brain, Cognition and Behaviour, Geert Grootplein Noord 21, Nijmegen, 6525 EZ The Netherlands

## Abstract

Mutations of *FOXP2* in 7q31 cause a rare disorder involving speech apraxia, accompanied by expressive and receptive language impairments. A recent report described a child with speech and language deficits, and a genomic rearrangement affecting chromosomes 7 and 11. One breakpoint mapped to 7q31 and, although outside its coding region, was hypothesised to disrupt *FOXP2* expression. We identified an element 2 kb downstream of this breakpoint with epigenetic characteristics of an enhancer. We show that this element drives reporter gene expression in human cell-lines. Thus, displacement of this element by translocation may disturb gene expression, contributing to the observed language phenotype.

Mutations and chromosomal rearrangements that disrupt the *FOXP2* coding sequencing cause childhood apraxia of speech (CAS) [also known as developmental verbal dyspraxia (DVD)], as well as expressive and receptive deficits in both spoken and written language [1–10]. Moralli et al. recently described a child with a complex chromosomal rearrangement affecting chromosome 7 and 11, showing severe speech and language problems, similar to the profile typically seen for *FOXP2* mutation cases [11]. For a detailed description of the phenotype we refer to the original clinical report [11].

The rearrangement in this child consists of a pericentric inversion of chromosome 7 (involving 7p15 and 7q31) and a translocation between chromosomes 7 and 11 (involving 7q21 and 11p12) [11]. The inversion and translocation breakpoints do not interrupt the sequence of any protein-coding genes [11]. It is therefore likely that the observed phenotype is caused by altered expression of nearby genes. *FOXP2* was considered the most promising candidate gene, given that haploinsufficency of this gene is known to cause speech and language disorders with a similar phenotype [8]. The chromosome 7q31 breakpoint was mapped to a position 205 kb downstream of the *FOXP2* locus and 22 kb upstream of the *MDFIC* gene (Fig. 1a). *MDFIC* expression was not significantly different in fibroblasts taken from the proband as compared to those from unaffected relatives. However, Moralli et al. were not able to reliably determine if the breakpoint affected *FOXP2* regulation, because this gene shows very low expression in fibroblasts.

**Fig. 1:**
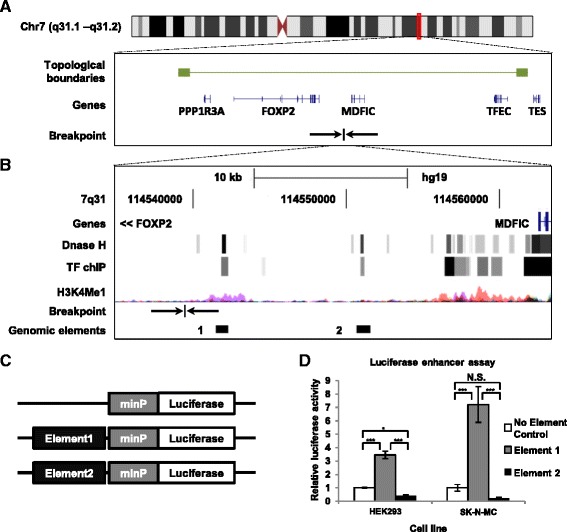
Identification of functional enhancer elements downstream of the inversion breakpoint. **a** The genomic location of the inversion breakpoint on chromosome 7q31 with the boundaries of the topological domain shown by green boxes [18]. Genes are depicted in blue. **b** Detailed view of the breakpoint with functional annotations from the ENCODE project [12] and UCSC genome browser [20]. The inversion breakpoint was mapped to position 114539340 of chromosome 7 in human genome build hg19 [11] (indicated by arrows). DNase hypersensitivity sites (DNase H) indicates regions of open chromatin. The darkness of the grey boxes is proportional to maximum signal strength of the DNase hypersensitivity in any investigated cell line. TF ChIP indicates sites that were occupied by TFs and the colour intensity is proportional to the amount of different TFs found at one site. The H3K4Me1 track indicates the presence of histone-3 lysine-4 mono-methylation in human cell lines. Pink peaks correspond to those found in NHEK cells (colorectal carcinoma) and orange peaks to those found in GM12878 (EBV transformed lymphoblast) cells. The investigated genomic elements are represented as black bars. Element 1 overlaps a DNase H site detected in 48 cell lines and 4 TFs. Element 2 overlaps a DNase H site detected in 17 cell lines and 1 TF. **c**-**d** Reporter constructs containing no enhancer (no element control), Element 1 (chr7: 114541370 – 114542201, hg19) and Element 2 (chr 7: 114550520 – 114551429, hg19) were transfected into HEK293 and SK-N-MC cells. After 2 days the luciferase activity was determined as described before [21]. The firefly luciferase activity was normalized to the activity of a co-transfected renilla luciferase control to determine the relative luciferase activity. The luciferase activity driven by the genomic elements was compared to that obtained from the control luciferase plasmid (minP). Element 1, but not Element 2 was able to enhance the activity and therefore the expression of the reporter gene. We performed two independent experiments of each three biological replicates. Significance was determined by an ANOVA followed by post-hoc Tukey test.*p < 0.05, ***p < 0.001. N.S.: not significant

We hypothesized that the inversion in the Moralli et al. case would physically separate the *FOXP2* coding region from a genomic element with the potential to regulate expression of this gene. In this letter we identify and characterize a functional regulatory element located >205 kb downstream of *FOXP2*. Our findings suggest a mechanism by which the breakpoint could disrupt regulation of *FOXP2* expression and provide support for the causative nature of this rearrangement.

To determine if the 7q31 breakpoint disrupted a regulatory element we used functional genomics data from the ENCODE project [12] to predict possible enhancer regions that would drive gene expression. Although the reported breakpoint did not directly disrupt any predicted enhancers, we identified two possible enhancers that are in close proximity. One element, located 2.5 kb downstream of the breakpoint (Element 1), includes a region of open chromatin (demonstrated via DNase hypersensitivity across multiple cell lines) and carries histone modifications characteristic of an enhancer (H3K4Me1) [13, 14] (Fig. 1b). This genomic site has been shown to bind several transcription factors (TF) in a colorectal carcinoma cell line (TF ChIP; Fig. 1b), including RNA polymerase II, which is found at transcriptional start sites and active enhancers [15, 16]. A second candidate region, located 12 kb downstream of the breakpoint (Element 2), shows DNase hypersensitivity and TF binding, but no H3K4Me1 modifications (Fig. 1b).

To test whether these regions could act as enhancer elements we cloned them into a reporter construct in front of a minimal promoter and luciferase reporter gene (Fig. 1c). The resulting constructs were used to measure the ability of each element to drive increased expression of the reporter gene in two human cell-lines; HEK293 and SK-N-MC. Since both cell lines endogenously express *FOXP2* [17] they likely express TFs that are able to regulate this gene. Element 1, which had multiple chromatin signatures characteristic of an enhancer, was able to act as a functional enhancer in both cell-lines (Fig. 1d). In HEK293 cells we observed a 3 fold increase of luciferase expression in comparison to the empty vector control. In SK-N-MC cells the luciferase expression increased nearly 7 fold as compared to the control. Element 2, which lacked histone modifications characteristic of an enhancer, was not able to drive expression in either cell line. Therefore, Element 1 is a functional enhancer capable of driving increased gene expression in both human cell lines.

Thus, following fine-mapping of the breakpoint by Moralli et al. [11], we were able to identify an active enhancer that is displaced by the chromosome 7 inversion. Genome wide structural mapping has shown that there are topological boundaries that regulatory elements are unlikely to cross [18]. Enhancers usually regulate genes that lie within the same topological domain, suggesting 4 genes to be potentially regulated by Element 1; *PPP1R3A, FOXP2, MDFIC* and *TFEC.* The inversion separates Element 1 from *PPP1R3A* and *FOXP2.* Given that *PPP1R3A* is a muscle specific gene not thought to be expressed in the brain [19], we consider the disrupted regulatory control of *FOXP2* likely to be a contributing factor to the phenotype found in this proband.

In sum, our functional data provide experimental support to the theory posited in Moralli et al. [11] that the chromosome 7 breakpoint carried by this patient contributed to the speech and language phenotype by disrupting the regulation of *FOXP2*.

## Abbreviations

DVD: Developmental verbal dyspraxia
CAS: Childhood apraxia of speech
Kb: Kilo base
H3K4Me1: Histone-3 lysine-4 mono-methylation
TF: Transcription factor
N.S.: Not significant

## References

[1] Lai CS, Fisher SE, Hurst JA, Vargha-Khadem F, Monaco AP (2001). A forkhead-domain gene is mutated in a severe speech and language disorder. Nature.

[2] MacDermot KD, Bonora E, Sykes N, Coupe AM, Lai CS, Vernes SC (2005). Identification of FOXP2 truncation as a novel cause of developmental speech and language deficits. Am J Hum Genet.

[3] Shriberg LD, Ballard KJ, Tomblin JB, Duffy JR, Odell KH, Williams CA (2006). Speech, prosody, and voice characteristics of a mother and daughter with a 7;13 translocation affecting FOXP2. J Speech Lang Hear Res.

[4] Feuk L, Kalervo A, Lipsanen-Nyman M, Skaug J, Nakabayashi K, Finucane B (2006). Absence of a paternally inherited FOXP2 gene in developmental verbal dyspraxia. Am J Hum Genet.

[5] Zeesman S, Nowaczyk MJ, Teshima I, Roberts W, Cardy JO, Brian J (2006). Speech and language impairment and oromotor dyspraxia due to deletion of 7q31 that involves FOXP2. Am J Med Genet A.

[6] Zilina O, Reimand T, Zjablovskaja P, Mannik K, Mannamaa M, Traat A (2012). Maternally and paternally inherited deletion of 7q31 involving the FOXP2 gene in two families. Am J Med Genet A.

[7] Palka C, Alfonsi M, Mohn A, Cerbo R, Guanciali Franchi P, Fantasia D (2012). Mosaic 7q31 deletion involving FOXP2 gene associated with language impairment. Pediatrics.

[8] Rice GM, Raca G, Jakielski KJ, Laffin JJ, Iyama-Kurtycz CM, Hartley SL (2012). Phenotype of FOXP2 haploinsufficiency in a mother and son. Am J Med Genet A.

[9] Laffin JJ, Raca G, Jackson CA, Strand EA, Jakielski KJ, Shriberg LD (2012). Novel candidate genes and regions for childhood apraxia of speech identified by array comparative genomic hybridization. Genet Med.

[10] Turner SJ, Hildebrand MS, Block S, Damiano J, Fahey M, Reilly S (2013). Small intragenic deletion in FOXP2 associated with childhood apraxia of speech and dysarthria. Am J Med Genet A.

[11] Moralli D, Nudel R, Chan MT, Green CM, Volpi EV, Benitez-Burraco A (2015). Language impairment in a case of a complex chromosomal rearrangement with a breakpoint downstream of FOXP2. Mol Cytogenet.

[12] ENCODE (2012). An integrated encyclopedia of DNA elements in the human genome. Nature.

[13] Hon GC, Hawkins RD, Ren B (2009). Predictive chromatin signatures in the mammalian genome. Hum Mol Genet.

[14] Shlyueva D, Stampfel G, Stark A (2014). Transcriptional enhancers: from properties to genome-wide predictions. Nat Rev Genet.

[15] Louie MC, Yang HQ, Ma AH, Xu W, Zou JX, Kung HJ (2003). Androgen-induced recruitment of RNA polymerase II to a nuclear receptor-p160 coactivator complex. Proc Natl Acad Sci U S A.

[16] Bonn S, Zinzen RP, Girardot C, Gustafson EH, Perez-Gonzalez A, Delhomme N (2012). Tissue-specific analysis of chromatin state identifies temporal signatures of enhancer activity during embryonic development. Nat Genet.

[17] Schroeder DI, Myers RM (2008). Multiple transcription start sites for FOXP2 with varying cellular specificities. Gene.

[18] Dixon JR, Selvaraj S, Yue F, Kim A, Li Y, Shen Y (2012). Topological domains in mammalian genomes identified by analysis of chromatin interactions. Nature.

[19] Tang PM, Bondor JA, Swiderek KM, DePaoli-Roach AA (1991). Molecular cloning and expression of the regulatory (RG1) subunit of the glycogen-associated protein phosphatase. J Biol Chem.

[20] Kent WJ, Sugnet CW, Furey TS, Roskin KM, Pringle TH, Zahler AM (2002). The human genome browser at UCSC. Genome Res.

[21] Deriziotis P, O’Roak BJ, Graham SA, Estruch SB, Dimitropoulou D, Bernier RA (2014). De novo TBR1 mutations in sporadic autism disrupt protein functions. Nat Commun.

